# Continuous Full-Domain Highway Trajectory Tracking Based on Improved Deep-SORT and Inverse Covariance Intersection

**DOI:** 10.3390/s26134251

**Published:** 2026-07-04

**Authors:** Zheye Tian, Changhuizi Duan, Shijie Gao, Jianling Gu, Nengchao Lyu

**Affiliations:** 1 Hebei Expressway Group Co., Ltd., Jingqin Branch, Qinhuangdao 066001, China; 13832488886@126.com (Z.T.); 13831713999@163.com (S.G.); 2 Intelligent Transportation Systems Research Center, Wuhan University of Technology, Wuhan 430063, China; dchz@whut.edu.cn; 3 Hebei Expressway Group Co., Ltd., Shijiazhuang 050035, China; gujianling1983@163.com

**Keywords:** traffic engineering, vehicle trajectory tracking, multisensor fusion, trajectory stitching

## Abstract

**Highlights:**

**What are the main findings?**

- We develop a two-stage “object-level–decision-level” fusion model and an improved Deep-SORT algorithm incorporating an occluded target tracking controller (OTTC), achieving a multi-object tracking accuracy of over 90% in complex traffic.
- We propose a geometry-motion consistency stepwise calibration (GMCSC) method alongside an inverse covariance intersection (ICI)-based trajectory stitching strategy, yielding a 98.4% identity association accuracy for cross-domain trajectories.

**What are the implications of the main findings?**

- The proposed multimodal fusion and cross-domain stitching paradigm effectively mitigates the spatiotemporal fragmentation inherent to unimodal roadside perception, enabling scalable and uninterrupted macroscopic traffic flow observation.
- By synthesizing continuous, high-fidelity, full-domain vehicle trajectories, this framework provides trajectory data support for subsequent traffic safety analysis, risk-evolution studies, and complex traffic-flow dynamics analysis.

**Abstract:**

Continuous full-domain vehicle trajectories are essential for smart highway monitoring, but single-sensor roadside perception is limited by physical coverage, occlusion, and environmental sensitivity. To address continuous trajectory tracking across multiple roadside-sensing domains, this study proposes a real-time, full-domain highway trajectory tracking framework based on radar–camera fusion, improved Deep-SORT, and inverse covariance intersection. At the local perception level, a two-stage object-level and decision-level fusion model is constructed, and Deep-SORT is improved using a CIoU matching strategy and an occluded target tracking controller to enhance local multi-object tracking continuity. At the cross-domain association level, a geometry-motion consistency stepwise calibration method is developed to unify adjacent sensing domains, and a CATS-ICI trajectory stitching strategy is introduced to improve trajectory association and state smoothness during sensor handover. The proposed framework was validated on a real highway test section with roadside radar, video, and drone-based ground-truth trajectories. Experimental results show that the full local method achieves an EMOTA of 92.35%, and the reconstructed full-domain trajectories achieve a successful trajectory matching rate of 98.4% under the 452 vehicles/10 min test condition. Additional ablation experiments further verify the contributions of radar–camera fusion, CIoU, OTTC, GMCSC, CATS, and ICI. These results demonstrate that the proposed framework can provide continuous and reliable full-domain vehicle trajectories for real-world highway monitoring.

## 1. Introduction

With the accelerating advancement of transportation system intelligence, smart highway development centered on traffic digital twins has gained substantial momentum [1]. As the foundational layer for traffic state perception, large-scale roadside sensor networks play a pivotal role in acquiring dynamic traffic information. High-precision traffic perception not only provides an objective representation of the operational state of the physical traffic system, but also supplies a critical data foundation for refined road management, including dynamic risk assessment [2], high-risk event prediction [3], and trajectory-based traffic safety analysis [4]. Therefore, establishing a roadside perception system with full spatial coverage of the roadway, capable of accurately extracting vehicle trajectory data using multimodal sensors [5], is a fundamental prerequisite for realizing transportation digital twins and proactive safety management.

At present, vehicle trajectory data are primarily collected using cameras, infrared thermal cameras, LiDAR, and millimeter-wave radar. Among these, target tracking technology based on machine vision is the most widely applied [6]. Since the introduction of the scale-invariant feature transform (SIFT) [7], a series of efficient feature extraction algorithms, including SURF and ORB, have been developed, markedly improving the stability of visual localization. Building on advances in vision-based perception, several open-source vehicle trajectory datasets have been established, including HighD, InD, RounD, and NGSIM. However, owing to the physical sensing boundaries and environmental sensitivity of single-sensor vision systems, such as cameras and unmanned aerial vehicles [8], existing datasets generally suffer from limited observation range (typically with a maximum coverage radius of less than 640 m), insufficient temporal continuity (continuous observation durations of less than 12 h), and weak scene generalizability, particularly under adverse weather conditions and in complex environments such as tunnels [9,10]. These limitations hinder their ability to support large-scale traffic-flow observation over wide spatial extents and long temporal horizons [11].

Recent studies have explored vehicle state estimation and trajectory acquisition using single-sensor perception. Xu and Liu [12] proposed a keypoint detection-based and multi-deep learning model integrated method to identify the spatial-temporal distribution of vehicle axle loads from monocular vision, demonstrating the potential of camera-based methods for vehicle spatial state estimation under favorable imaging conditions. However, single-sensor perception is still constrained by physical observation boundaries and environmental sensitivity. Camera-only methods are susceptible to camera pose, calibration quality, target scale variation, occlusion, illumination, and visibility, especially under rain, fog, low illumination, and nighttime conditions. Previous nighttime vehicle detection research based on image translation technology has further shown that low-light conditions can significantly affect visual perception even when enhancement strategies are adopted [13]. To alleviate these limitations, Li et al. [14] developed RVIFNet to fuse radar and visual information for all-weather vehicle perception in roadside monitoring scenarios, showing that camera-based semantic and contour information can be complemented by radar-based range and velocity measurements. Xiang et al. [15] further reviewed multisensor fusion and cooperative perception methods and emphasized their value in improving object-tracking reliability in complex environments. Nevertheless, existing multisensor fusion studies mainly focus on object detection or trajectory tracking within a local single-scene sensing area, and they do not fully address continuous full-domain tracking across multiple roadside-sensing domains.

Due to the constraints imposed by complex traffic environments and traffic-flow characteristics, existing studies on vehicle trajectory extraction have mainly focused on single-scene settings, and methods for global continuous trajectory extraction remain scarce. In terms of roadside radar perception, although multi-radar cascading and stitching can reconstruct trajectories over specific road sections [16], its generalizability under dense traffic flow and occlusion conditions has yet to be fully validated. In terms of roadside vision, cross-domain trajectory stitching techniques have shown good performance on urban roads with favourable top-down viewing angles [17]. However, in highway scenarios, high vehicle speeds and insufficient pitch angles caused by the height limits of standard roadside poles lead to severe occlusion, substantially increasing the difficulty of continuous tracking and matching across multiple cameras [18,19]. Moreover, roadside sensors are continuously exposed to outdoor environments and are vulnerable to disturbances such as strong winds, rain and snow, and vibrations induced by heavy vehicles, which may cause sensor pose drift and calibration-parameter deviations. These factors further increase the difficulty of maintaining consistent spatial mapping and reliable trajectory association across sensing domains. Therefore, compared with existing studies that mainly focus on local detection, single-scene tracking, or direct trajectory stitching, continuous full-domain highway tracking still requires a unified framework that can jointly address cross-domain calibration, ambiguous trajectory association, and state smoothing during sensor handover.

To address the above challenges and meet the practical requirements of highway deployment, this study proposes a real-time full-domain vehicle trajectory tracking method based on roadside multisensor fusion. At the local perception level, a two-stage object-level and decision-level fusion model is constructed based on AdaBoost and Dempster–Shafer evidence theory, and Deep-SORT is improved to reduce trajectory interruption caused by vehicle occlusion, thereby achieving continuous local multi-object tracking. At the full-domain association level, a geometry-motion consistency stepwise calibration (GMCSC) method is proposed to refine the spatial mapping between adjacent sensing domains, and a trajectory stitching strategy combining bidirectional selection matching with inverse covariance intersection (ICI)-based state fusion is designed. Unlike methods that mainly rely on fixed geometric parameters or a single trajectory-similarity cue, GMCSC jointly uses road geometry similarity and vehicle motion consistency, while the CATS-ICI strategy integrates clue-aware trajectory matching with recursive state fusion. In this way, the proposed framework improves cross-domain association stability, suppresses handover-induced trajectory jitter, and supports smooth reconstruction of full-domain vehicle trajectories.

## 2. Local Vehicle Detection Fusion Algorithm

To satisfy the requirement for high-precision vehicle perception within the coverage range of a single roadside sensing unit, this section proposes a local-scene vehicle detection and tracking fusion framework based on heterogeneous sensor data. First, a two-stage information fusion model, consisting of object-level fusion and decision-level fusion, is constructed. The DIoU and AdaBoost algorithms are used to accurately determine target association states, while Dempster–Shafer evidence theory is incorporated to improve the confidence of vehicle detection under complex environmental conditions. Finally, an improved Deep-SORT algorithm is introduced. By designing a CIoU-based matching strategy and an occluded target tracking controller (OTTC), the proposed method effectively addresses trajectory interruption caused by local vehicle occlusion and nonlinear motion, thereby achieving high-precision continuous multi-object tracking in the local sensing area.

### 2.1. Single-Target Fusion Algorithm

Vehicle operating states on highways are influenced by factors such as traffic flow characteristics, vehicle type composition, and occlusion by surrounding objects. As a result, vehicle detection data obtained from a single sensor often suffer from substantial measurement errors and missed detections, making it difficult to satisfy the accuracy requirements of vehicle tracking. It is therefore necessary to further fuse vehicle information detected by multiple sensors. Considering the requirement of real-time monitoring, this study adopts a decision-level fusion algorithm to associate and fuse vehicle data detected by radar and camera sensors.

**Figure 1:**
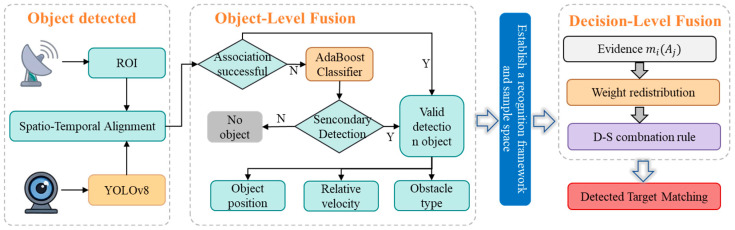
Two-stage information fusion framework for camera and radar.

A two-stage information fusion model integrating object-level fusion and decision-level fusion was developed in this study, as shown in Figure 1. The model is built on millimeter-wave radar and camera sensors and consists of four main components: sensor data acquisition and processing, object-level fusion, decision-level fusion, and fused output generation. First, a parallel fusion scheme is adopted, in which the millimeter-wave radar is used to measure the distance to targets ahead, while the camera is mainly used for target detection, producing two types of vehicle detection information. Next, the two types of information are matched and associated. The association result is compared with a predefined threshold to determine whether secondary detection should be performed using the AdaBoost algorithm. The object-level fusion results are then used as the input for decision-level fusion. Finally, Dempster–Shafer evidence theory is applied to obtain the final target category and driving-state information, including relative position. The specific algorithm steps are as follows:

(1) Construction of the association matrix. Most multisensor data association algorithms perform matching based on the ranking of Euclidean distances, such as the nearest neighbor (NN) and global nearest neighbor (GNN) methods. These algorithms are simple, intuitive, and relatively easy to implement. However, ranking based solely on Euclidean distance considers only the absolute distance between two target points in a two-dimensional image, without accounting for the spatial distribution of regions of interest or the relationships between target features. As a result, the matching performance is often unsatisfactory, and the association error may also be large. Therefore, this study adopts DIoU (Distance-IoU) [20] to construct an association matrix based on both the overlap ratio of detection boxes and the normalized Euclidean distance between their center points. The specific formulation is as follows:

(1) $$\left\{\begin{array}{l} IoU_{ij}=\frac{\left|C_{i}\cap R_{j}\right|}{\left|C_{i}\cup R_{j}\right|}\\ d_{ij}=\sqrt{\left(u_{i}-u_{j})+(v_{i}-v_{j}\right)}\\ D_{ij}=1-\frac{d_{ij}^{2}}{c_{ij}^{2}}\\ P=1-[\alpha IoU+(1-\alpha )D] \end{array}\right.$$

where *i* and *j* denote the *i*-th target detected by the camera and the *j*-th target detected by the radar, respectively; *C* and *R* denote the areas of the camera detection box and the radar-projected detection box, respectively; $IoU_{ij}$ denotes the overlap ratio between the two detection boxes; $d_{ij}$ denotes the Euclidean distance between the pixel coordinates of the two target centers; $c_{ij}$ denotes the diagonal length of the minimum enclosing rectangle covering the two detection boxes; *P* denotes the association matrix after normalization and weighted averaging; and α is the weighting coefficient.

**Figure 2:**
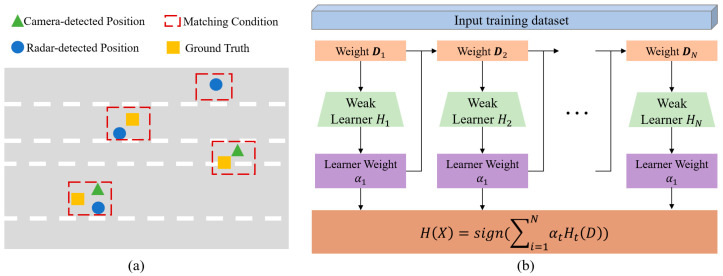
Perceived target association process. (**a**) Association states among radar, camera, and ground-truth targets. (**b**) AdaBoost ensemble classification process for target association.

(2) Target association algorithm. Within the same frame, the association status of a vehicle target detected by different sensors can be classified into four categories: (1) the target is detected by both the vision sensor and the millimeter-wave radar; (2) the target is detected only by the vision sensor; (3) the target is detected only by the millimeter-wave radar; and (4) a false target is detected by either the camera or the radar, as shown in Figure 2a. For different experimental scenarios, the AdaBoost algorithm is adopted in this study to analyze the association results. This algorithm trains a series of weak classifiers by dynamically adjusting sample weights. In each iteration, the weights of correctly classified samples are decreased, whereas those of misclassified samples are increased, so that subsequent classifiers focus more on samples that are difficult to classify. The updated training samples are then input into the next classifier layer for further training, thereby gradually improving the classification accuracy. Finally, the weak classifiers obtained in each round are combined through weighted aggregation to construct a strong classifier as the final decision model. The overall procedure of the algorithm is shown in Figure 2b.

(3) Target fusion algorithm (TFA). The object-level fusion results are used as the input to the decision-level fusion stage. Dempster–Shafer (D–S) evidence theory is then applied to reassign the basic probability masses of the evidence obtained from object-level fusion. Let the frame of discernment be defined as $\Theta =\{A_{1},A_{2},\ldots ,A_{n}\}$, and let $M_{i}=\{m_{i}(A_{1}),m_{i}(A_{2}),\ldots ,m_{i}(A_{n})\}$ denote the evidence vector, where the element $M_{i}$ represents the confidence assigned by sensor *i* to target class *j*. The evidence set is therefore expressed as $M=\{M_{1},M_{2},\ldots ,M_{n}\}$. The deviation between two evidence vectors is described by constructing a distance measure between them. In this study, the Pearson correlation coefficient is adopted to characterize the distance between two evidence vectors. Compared with the commonly used cosine similarity, the Pearson correlation coefficient can avoid error interference caused by simple vector translation and can better measure the difference between two individual vectors in the feature space. Under the frame of discernment Θ, the distance $d_{ab}$ between evidence $M_{a}$ and evidence $M_{b}$ is defined as follows:

(2) $$\left\{\begin{array}{l} d_{ab}=e^{1-P(M_{a},M_{b})}\\ P(M_{a},M_{b})=\frac{Cov(M_{a},M_{b})}{\sqrt{Var[M_{a}]\times Var[M_{b}]}} \end{array}\right.$$

According to the principle of mathematical induction, the conflict between the other pieces of evidence and evidence $M_{a}$ can be expressed as $con(M_{a})=\sum_{a=1,a\neq b}^{m}d_{ab}$. An evidence weight allocation coefficient is then defined as $\lambda (M_{a})=[1-in(M_{a})]e^{-in(M_{a})}$ to describe the importance of the information corresponding to evidence $M_{a}$ in the fusion process. If the original basic probability assignment is $m_{a}(A_{b})$, the reassigned basic probability is updated as $m_{a}^{*}(A_{b})=\lambda (M_{a})m_{a}(A_{b})$. Finally, the reassigned basic probability values are substituted into the D–S evidence theory combination rule for fusion decision-making.

### 2.2. Multi-Object Tracking Algorithm

**Figure 3:**
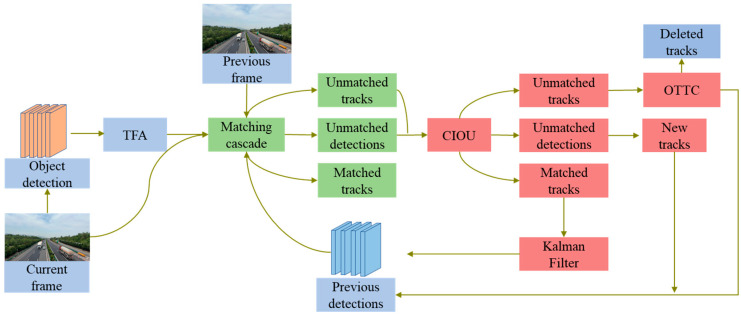
Flowchart of the improved Deep-SORT algorithm.

After accurate vehicle positions are obtained for each frame through decision-level fusion, an improved Deep-SORT algorithm is employed to match the fused appearance and motion information in order to maintain target consistency during continuous local tracking and complete the multi-object tracking task. The improved Deep-SORT algorithm is designed to address target recovery after occlusion, the re-identification of targets under pose variation, and the nonlinear prediction of target motion in complex dynamic environments. It mainly consists of three components: target detection, target matching, and trajectory prediction. In the target-matching stage, a CIoU-based matching algorithm is introduced to improve target re-identification accuracy in complex dynamic scenes. In the trajectory prediction stage, an occluded target tracking controller (OTTC) is proposed to enhance the trajectory tracking capability of the algorithm under complex dynamic conditions. The overall workflow of the algorithm is illustrated in Figure 3.

(1) CIoU matching algorithm. In the IoU matching stage of Deep-SORT, only the maximum overlap between the detection box and the predicted box is considered, while the similarity between the bounding boxes and the consistency and continuity of the target motion direction are ignored. To address this issue, a complete IoU (CIoU) calculation method is proposed. In addition to considering the shape similarity between the two bounding boxes, as measured by the center distance ($\rho_{a,b}$) between the detection box and the predicted box and the diagonal distance ($\delta_{a,b}$), the proposed CIoU method also introduces the concept of matching direction. Specifically, the parallelism loss is calculated based on the matching direction vector $v_{1}$ at time *t* − 1 and the direction vector $v_{2}$ at time *t*, so as to evaluate the directional consistency between the two matching results.

(3) $$\left\{\begin{array}{l} CIOU=IOU-\frac{\rho_{a,b}^{2}}{\delta_{a,b}^{2}}-Loss_{parallel}\\ Loss_{parallel}=1-a\cos\frac{v_{1}v_{2}}{\left|v_{1}\right|\left|v_{2}\right|} \end{array}\right.$$

(2) Occluded target tracking controller. For complex dynamic traffic environments containing a large number of occluded targets, an occluded target tracking controller (OTTC) is designed to preserve target trajectories as completely as possible. The specific procedure is as follows. (1) The trajectory set $Tra_{block}$, whose targets are identified as occluded during the second matching stage, is input into the OTTC. (2) All trajectories in $Tra_{block}$ are traversed, and the number of missed detections $N_{miss}$ for each trajectory is incremented by 1. If $N_{miss}>30$, the trajectory is removed from $Tra_{block}$. (3) If $N_{miss}\leq 30$, the trajectory at the next time step is predicted, and all qualified trajectories $Tra_{match}$ in $Tra_{block}$ are updated. (4) A new tracking set $Tra_{all}$ is initialized, and all qualified trajectories $Tra_{match}$ obtained in Step 3, together with the trajectories successfully matched in the second matching stage, are added to the new tracking set.

## 3. Full-Domain Trajectory Tracking for Continuous Roadway Monitoring

Building on stable local trajectories, we propose a collaborative trajectory-stitching algorithm for continuous multi-site roadway monitoring scenarios to overcome the physical sensing limitations of a single sensor group. The algorithm mainly addresses two key challenges: the unification of spatial reference frames across sensors and the association of trajectories in handover blind zones. On the one hand, cross-domain calibration is formulated as an optimization problem. A stepwise collaborative optimization strategy is proposed based on both road geometry similarity and the consistency of vehicle kinematics in overlapping regions, thereby enabling accurate mapping of cross-domain sensors into a unified global coordinate system. This design can partially reduce the influence of small installation or calibration deviations on cross-domain spatial mapping, although large pose changes or insufficient overlapping regions may still degrade association performance. On the other hand, to address ambiguous trajectory association in overlapping areas, a bidirectional selection matching method based on clue-aware trajectory similarity (CATS) [21] is developed. After successful association, the inverse covariance intersection (ICI) algorithm is introduced to recursively fuse the global vehicle states. This mechanism effectively alleviates observation jitter during continuous radar handover and ensures the spatial smoothness and temporal continuity of full-domain vehicle trajectories.

### 3.1. Cross-Domain Collaborative Sensor Calibration Method

The essence of sensor calibration is to establish an accurate mapping from the local radar coordinate system to the road coordinate system. As emphasized in previous taxonomy and analysis of camera calibration methods for traffic monitoring applications, calibration quality and camera deployment parameters have a direct influence on the reliability of roadside traffic perception [22]. Within the sensor coverage range, because highway alignment is generally smooth, the local coordinate system can be regarded as a small segment of the road curvilinear coordinate system. The objective of sensor calibration is therefore to determine a transformation matrix $H_{G}$ that satisfies the relation $\prod =\Psi H_{G}$, where ∏ denotes the trajectory data in the target road coordinate system and Ψ represents the set of raw data points measured by the sensor. Since the sensor position parameter $c_{1}$ is fixed, the calibration process considers only the rotation angle $\theta_{n}$ and the longitudinal scaling coefficient $\alpha_{n}$.

**Figure 4:**
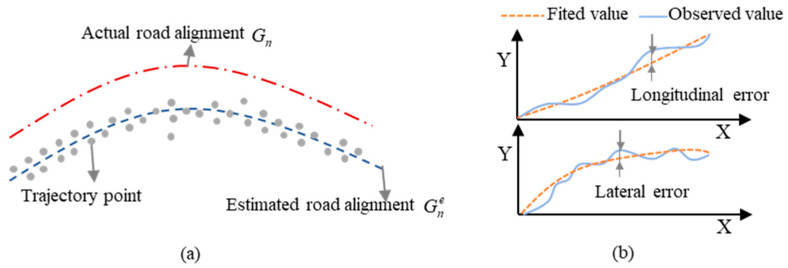
Stepwise calibration algorithm based on road geometry and motion consistency. (**a**) Geometry-consistency alignment based on road-alignment deviation. (**b**) Motion-consistency alignment based on trajectory residuals in the overlapping region.

To estimate the transformation matrix $H_{G}$, it is necessary to construct the corresponding objective function. For the calibration of continuously deployed sensors, this study proposes two core optimization cues: road geometry similarity and vehicle motion consistency in the overlapping region. Road geometry similarity is quantified by the variance $L_{1}$ between the estimated curve $G_{n}^{e}$ and the actual geometry $G_{n}$, as shown in Figure 4a. The problem of vehicle kinematic consistency in the overlapping region is transformed into a minimum-residual problem, denoted by $L_{2}$, between the fitted polynomial trajectory and the trajectory points, as shown in Figure 4b. Since both cues rely on stable road alignment and continuous vehicle motion patterns, the proposed GMCSC method is more suitable for highway sections with relatively smooth horizontal geometry. For road sections with sharp curvature changes or obvious elevation variations, additional elevation information, three-dimensional road geometry, or segment-wise local calibration may be required to further reduce mapping errors.

The optimization of the rotation angle $\theta_{n}$ and the longitudinal scaling coefficient $\alpha_{n}$ are performed using a stepwise optimization strategy. The core idea of this strategy is to guide the optimization sequence using physical prior knowledge: the objective function is first approached within the large-gradient range corresponding to the rotation angle by adjusting the radar rotation parameter $\theta_{n}$. Then, fine-tuning is carried out within the small-gradient range corresponding to longitudinal scaling in order to optimize the potential deviation of the radar scaling coefficient $\alpha_{n}$. The initial rotation angles, $\theta_{1}$ and $\theta_{2}$, are determined from the nominal installation orientations of adjacent roadside sensors relative to the local road tangent direction, and the initial longitudinal scaling coefficient is set to $\alpha_{s}$ = 1. The subsequent search is conducted within bounded intervals around these initial values. The detailed optimization procedure is presented in Algorithm 1.
**Algorithm 1** Geometry-Motion Consistency Stepwise Calibration (GMCSC)**Input:** Two sets of trajectory data **Output:** Optimized rotation parameters $\theta_{1}$, $\theta_{2}$; Scaling parameters $\alpha_{1}$ 1. Fix radar positions: Keep $c_{1}$ fixed 2. Initialize parameters: Set $\theta_{1}$ and $\theta_{2}$ according to the nominal sensor installation orientations and local road tangent direction, and set $\alpha_{s}$ = 1. 3. Step 1: Road Geometry Consistency Calibration 4. Construct error function $L_{1}=\underset{\theta_{n}}{\min}Var(G_{n}^{e}-G_{n})$ 5. Minimize $L_{1}$ with fixed $c_{1}$, and update $\theta_{n}$ 6. Step 2: Motion Consistency Calibration 7. After determining rotation parameters, extract trajectory pairs $Tra_{rm}^{i}$ in the overlapping area of the two radars 8. Fit a cubic polynomial $f_{i}(t)$ for the position differences of corresponding key points 9. Calculate residual error $L_{2}=\frac{1}{N_{i}}{\sum \left\|\left.f_{i}(t)-Tra_{rm}^{i}\right\|\right.}^{2}$ 10. Minimize $L_{2}$ with fixed $\theta_{n}$, and update $\alpha_{n}$ 11. return Final calibration parameters $\theta_{n}$, $\alpha_{n}$


The GMCSC optimization is performed within bounded physical parameter ranges determined by roadside sensor installation constraints, namely $\theta_{s}\in \left[\theta_{\min},\theta_{\max}\right]$ $\theta_{s}\in \left[\theta_{\min},\theta_{\max}\right]$ and $\alpha_{s}\in \left[\alpha_{\min},\alpha_{\max}\right]$. Since the input trajectory points are collected from a finite road section, their spatial coordinates are also bounded. For the motion-consistency residual $L_{2}=\frac{1}{N_{t}}\sum {\left|f(t)-Tra_{k}\right|}^{2}$, if the maximum spatial deviation within the feasible search range is denoted by $D_{\max}$, then each residual term satisfies $\left|f(t)-Tra_{k}\right|\leq D_{\max}$ and, therefore, $L_{2}\leq D_{\max}^{2}$. In addition, the stepwise search retains parameter updates that reduce the objective function, so the residual sequence satisfies $L_{2}^{k+1}\leq L_{2}^{k}$. Because $L_{2}$ is non-negative, the residual sequence is bounded below by 0 and converges to a finite value. Thus, the residual error remains bounded during iteration, and numerical overflow is avoided in the GMCSC implementation.

### 3.2. Cross-Domain Trajectory Association Algorithm

**Figure 5:**
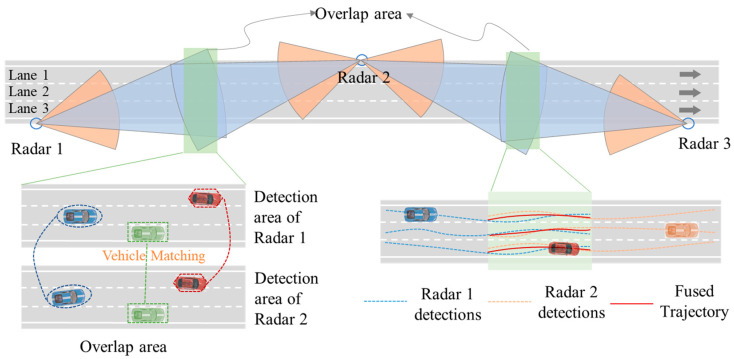
Overlapping and non-overlapping regions of the millimeter-wave radar group.

To ensure accurate merging of vehicle trajectories across different detection regions, the sensor coverage areas of adjacent observation points should include a certain degree of overlap, as shown in Figure 5. By associating the targets detected by the two sensor groups within the overlapping detection region, the same vehicle can be re-identified, and its trajectories can be merged across continuous multi-scene environments, thereby enabling continuous vehicle trajectory tracking.

The objective of trajectory association is to determine whether trajectories perceived by different sensors belong to the same target. To reduce ambiguous associations between trajectories detected by Sensor Group 1 and Sensor Group 2, we propose a bidirectional matching method that maximizes inter-trajectory similarity. Considering the spatial and temporal similarity of trajectories in the overlapping region, clue-aware trajectory similarity (CATS) [18] is adopted to evaluate trajectory similarity. Given a spatial threshold η and a temporal threshold τ, the CATS between trajectories $T_{i}$ and $T_{j}$ is defined as follows:

(4) $$\left\{\begin{array}{l} CATS_{\eta ,\tau }=\frac{1}{\left|T_{i}\right|}\times \sum_{\eta ,T_{j}\in T_{i}}score_{\eta ,\tau }(p_{i},\eta ,T_{j})\\ score(p_{i},\eta ,T_{j})=\max\{f_{e}(p_{i},\eta ,p_{j,k})|p_{j,k}\in T_{j}\}\\ f_{e}(p_{i},\eta ,p_{j,k})=\left\{\begin{array}{ll} 0 & ,if\;dist(p_{i,l},p_{j,k})>\eta \\ 1-\frac{dist(p_{i},\eta ,p_{j,k})}{e} & ,otherwise \end{array}\right. \end{array}\right.$$

where $p_{j,k}=(k_{j,k},t_{j,k})$ denotes the *k*-th trajectory point of the *j*-th trajectory and $p_{j,k}$ and $t_{j,k}$ represent the time and position of the trajectory point; dist(⋅,⋅) denotes the Euclidean distance between two points. It should be noted that $CATS_{\eta ,\tau }(T_{i},T_{j})$ is not necessarily equal to $CATS_{\eta ,\tau }(T_{j},T_{i})$.

Let ${\mathbb{Z}}^{1}$ and ${\mathbb{Z}}^{2}$ denote the sets of all trajectories detected by Sensor Group 1 and Sensor Group 2, respectively. For an arbitrary trajectory $T_{i}\in {\mathbb{Z}}^{1}$, its most similar trajectory is determined according to Equation (1), where γ is a predefined threshold. When CATS>γ, the two trajectories are considered to be associated.

(5) $$\left\{\begin{array}{l} S_{1\to 2}(T_{i}^{1})=\arg\max(CATS_{\eta ,\tau }(T_{i}^{1},T_{j}^{2}))\\ s.t.\;CATS_{\eta ,\tau }(T_{i}^{1},S_{1\to 2}(T_{i}^{1}))>\gamma \end{array}\right.$$

First, the posterior error covariance matrices of the sensors, denoted by $\sum_{1}$ and $\sum_{2}$, are obtained from the calibrated trajectory data. At the same time, the vehicle state vectors perceived by Radar 1 and Radar 2 at the same time instant are denoted by $x_{1}$ and $x_{2}$, respectively. The inverse covariance intersection (ICI) algorithm is then used to fuse the trajectory states. The recursive equations for the fused full-domain vehicle state vector $x_{f}$ and its covariance matrix $\sum_{f}$ are given as follows:

(6) $$\left\{\begin{array}{l} \sum_{f}={\left(\sum_{1}^{-1}+\sum_{2}^{-1}-{\left(\omega \sum_{1}+(1-\omega )\sum_{2}\right)}^{-1}\right)}^{-1}\\ \begin{array}{l} x_{f}=\sum_{f}(\sum_{1}^{-1}x_{1}+\sum_{2}^{-1}x_{2}-{\left(\omega \sum_{1}+(1-\omega )\sum_{2}\right)}^{-1}(\omega x_{1}+(1-\omega )x_{2}))\\ \omega =\arg\;\underset{\omega \in [0,1]}{\min}\;\mathrm{tr}[\sum_{f}] \end{array} \end{array}\right.$$

where ω∈[0,1] is the optimization weight parameter. To achieve optimal fusion performance, the weight ω is determined dynamically by minimizing the trace of the fused covariance matrix. The invertibility of $\sum_{f}$ is guaranteed when the corresponding information matrix $\sum_{f}^{-1}$ is non-singular. In the ICI implementation, the input covariance matrices $\sum_{1}$ and $\sum_{2}$ are required to be symmetric positive definite. Under ω∈[0,1], the weighted covariance matrix $\omega \sum_{1}+(1-\omega )\sum_{2}$ is also positive definite and therefore invertible. In practical computation, the positive definiteness of $\sum_{1}$ and $\sum_{2}$ is checked, and a small diagonal regularization term can be added when necessary to avoid numerical singularity. The boundedness of the fused state xf follows from the bounded input states and finite fusion matrices. Since the vehicle states $x_{1}$ and $x_{2}$ are obtained from a finite monitored road section within a finite observation time, their position and velocity components are bounded, i.e., $\left\|x_{1}\right\|\leq \rho_{1}$ and $\left\|x_{2}\right\|\leq \rho_{2}$. With positive definite covariance matrices and ω∈[0,1], the matrix coefficients in Equation (6) are finite. Therefore, there exist finite constants $c_{1}$ and $c_{2}$ such that $\left\|x_{f}\right\|\leq c_{1}\left\|x_{1}\right\|+c_{2}\left\|x_{2}\right\|\leq c_{1}\rho_{1}+c_{2}\rho_{2}=\rho$, where $\rho \in R_{+}$. Thus, the recursive fused state remains bounded and does not diverge. This fusion mechanism can effectively reduce observation jitter during continuous radar handover and significantly improve the smoothness and continuous localization accuracy of full-domain vehicle trajectories within the overlapping region.

## 4. Results Analysis

### 4.1. Experimental Design

**Figure 6:**
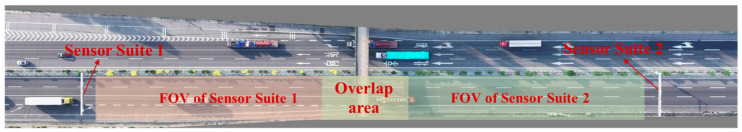
Layout of the test scenario.

To evaluate the performance of the proposed method, a continuous section of a highway was selected as the test scenario for coordinated data collection, manual annotation, and performance analysis. The study site is shown in Figure 6. The experimental road section had a total length of 450 m. Radar and video sensors were installed on the same roadside poles, and the distance between adjacent poles was 400 m. The overlap area of radar coverage exceeded 100 m. During radar and video data acquisition, a drone was simultaneously used to collect ground-truth trajectory data through aerial imaging.

The proposed framework was implemented on a deployable computing platform equipped with an Intel Core i9-12900KF CPU (Intel Corporation, Santa Clara, CA, USA), an NVIDIA GeForce RTX 4080 GPU (NVIDIA Corporation, Santa Clara, CA, USA), and 64 GB memory. The roadside camera operated at 30 fps, and the millimeter-wave radar operated at 10 Hz. For radar–camera fusion, the lower-frequency millimeter-wave radar data were used as the main sequence. After unifying the initial timestamps of the radar and video sequences, the visual sensor data were associated with the radar sequence to achieve time soft synchronization of the multisensor data, resulting in a synchronized fusion frequency of 10 Hz. The average processing times of camera-based detection, radar preprocessing, radar–camera association, and D–S evidence fusion improved Deep-SORT tracking, and CATS-ICI trajectory stitching were 18.6 ms, 0.9 ms, 1.7 ms, 5.8 ms, and 1.4 ms per synchronized cycle, respectively, resulting in a total processing time of 28.4 ms. This value is lower than the 100 ms sampling interval of the synchronized radar–camera data. The main computational burden came from visual detection and multi-object tracking, while the fusion and trajectory-stitching modules introduced only limited additional cost because they operate on compact target-level or trajectory-level data.

### 4.2. Analysis of Local Trajectory Fusion Performance

(1) Target detection accuracy

To evaluate the effectiveness of the proposed method, millimeter-wave radar data and roadside video detection data were used as benchmarks for comparison. For the evaluation of vehicle target detection accuracy, the detection range was divided into the near field (<100 m) and the far field (>100 m). The target detection accuracy was defined as $E_{n}=N_{n}/N_{GT}$, where $N_{GT}$ denotes the reference number of manually annotated trajectory points and $N_{n}$ denotes the number of detected trajectory points matched with the manual annotations. Based on this metric, the radar detection accuracy $E_{radar}$, video detection accuracy $E_{camera}$, and fused detection accuracy $E_{fused}$ were evaluated separately.

Table 1 compares the results of the proposed multisensor fusion algorithm with those of single-sensor detection methods. Overall, the proposed fusion algorithm achieved the best performance, demonstrating the effectiveness of the method. Specifically, compared with the single-sensor detection methods, the fusion algorithm improved detection performance by more than 5% in the near field and by nearly 10% in the far field. In addition, the fusion algorithm showed the most significant improvement under high-traffic conditions, with a performance gain of more than 10%.

(2) Trajectory tracking performance

On the basis of target detection accuracy, the MOTA-like tracking accuracy score $E_{MOTA}$ and multi-object tracking precision $E_{MOTP}$ were adopted to evaluate the accuracy of vehicle trajectory tracking:

(7) $$\left\{\begin{array}{l} E_{MOTA}=1-(E_{FP}+E_{FN}+E_{IDSW})/3\\ E_{MOTP}=\sum_{t}\sum_{i}D_{t,i}/\sum_{t}c_{t} \end{array}\right.$$

where $E_{FP}$ denotes the false-positive rate, $E_{FN}$ denotes the false-negative rate, $E_{IDSW}$ denotes the rate of identity switches, $D_{t,i}$ denotes the positional deviation, and $c_{t}$ denotes the number of successfully matched vehicles. It should be noted that $E_{MOTA}$ is a customized MOTA-like score rather than the standard MOTA.

**Figure 7:**
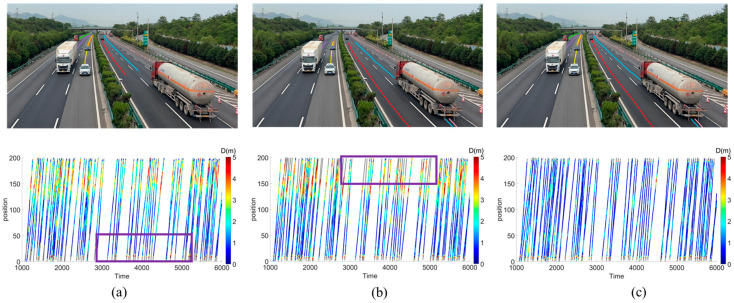
Vehicle trajectory tracking results under different detection methods. (**a**) Radar Perception; (**b**) Video Perception; (**c**) Fused Perception.

The upper panel of Figure 7 presents a visualization of the multi-object tracking trajectories obtained by the three detection methods from the camera view at the test site. Combined with the results in Table 1, the following observations can be made. Radar trajectories show a relatively high failure rate in the near field and are particularly prone to switching and fragmentation under dense traffic conditions because of limited lateral resolution. Vision-based trajectories are relatively complete in the near field, but trajectory detection tends to fail in the far field. In contrast, the fusion algorithm maintains complete trajectories throughout the detection range, indicating its superior detection performance.

Table 2 summarizes the local ablation results under different detection and tracking configurations. The comparison includes single-sensor baselines, radar–camera fusion with the original Deep-SORT tracker, fusion with the CIoU matching strategy, and the full local method with both CIoU and OTTC. The results show that radar–camera fusion improves the overall tracking accuracy, while CIoU and OTTC further reduce identity switches and improve trajectory continuity.

### 4.3. Analysis of Full-Domain Trajectory Tracking Performance in Continuous Monitoring

Post-processing was performed on the fused trajectories obtained from the continuous monitoring segment over the full domain. Based on the data in Table 3, system performance under different traffic volumes and vehicle-type compositions was evaluated from two aspects: successful trajectory matching rate (Match) and fused trajectory accuracy (MAE).

(1) Successful trajectory matching rate (Match).

The successful trajectory matching rate was defined as $R_{match}=(N_{total}-N_{unmatched})/R_{total}\times 100\%$, where $N_{total}$ denotes the total number of trajectories requiring cross-domain association and $N_{unmatched}$ denotes the number of unmatched trajectories. Under the test condition of 452 vehicles/10 min with a truck proportion of 15%, the successful trajectory matching rate of trajectories in the overlapping region reached 98.4%. As traffic volume increased to 761 vehicles/10 min and 956 vehicles/10 min, or as the truck proportion rose to 30%, the matching rate showed a downward trend because of increased mutual occlusion between vehicles. Even under the most complex test condition, with 956 vehicles/10 min and a truck proportion of 30%, the system still maintained a matching rate of 90.9%.

(2) Fused trajectory accuracy (MAE).

The mean absolute position error (MAE) of the fused trajectories increased with increasing traffic volume and truck proportion. Under the condition of 452 vehicles/10 min with a truck proportion of 15%, the error was 0.23 m. Under the demanding conditions with 956 vehicles/10 min and a truck proportion of 30%, the error increased to a maximum of 0.98 m.

**Figure 8:**
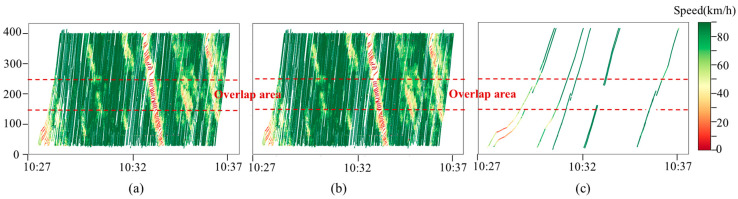
Comparison of full-domain fused trajectories and drone ground-truth trajectories. (**a**) Full-domain fused trajectories reconstructed by CATS-ICI. (**b**) Drone-derived ground-truth trajectories. (**c**) Local comparison between fused and ground-truth trajectories in the overlapping region.

Figure 8 presents a comparison between the fused trajectories and the drone-derived ground-truth trajectories. A total of 452 vehicle trajectories were observed over the full monitored area, among which only 7 trajectories were unmatched, as shown in Figure 8c, resulting in an overall trajectory completeness rate of 98.4% over the full monitored area. A comparison of Figure 8a,b shows that the perceived trajectories generated by the proposed algorithm agree closely with the ground-truth trajectories, with no obvious spatiotemporal deviation and only minor local discrepancies at sections with fluctuating traffic flow.

As shown in Table 4, removing GMCSC leads to a clear decrease in the successful trajectory-matching rate and an increase in MAE, indicating that cross-domain spatial calibration is essential for reliable trajectory association. Removing CATS also reduces the matching rate, which suggests that clue-aware trajectory similarity is more robust than simple spatiotemporal nearest-neighbor matching in ambiguous handover regions. When ICI is removed, the matching rate remains relatively high, but MAE increases, indicating that ICI mainly contributes to state smoothing and localization consistency during continuous sensor handover. The full method achieves the best performance, demonstrating the complementary contributions of GMCSC, CATS, and ICI.

## 5. Conclusions

To address the need for continuous and high-precision full-domain vehicle trajectory acquisition in highway scenarios, this study proposes a roadside radar–camera fusion framework integrating local multi-object tracking, cross-domain calibration, trajectory association, and state fusion.

(1) At the local perception level, the two-stage object-level and decision-level fusion model improves the reliability of vehicle detection, while the improved Deep-SORT with CIoU matching and OTTC enhances trajectory continuity under local occlusion. The full local method achieves an EMOTA of 92.35% in the real highway experiment.

(2) At the full-domain association level, the proposed GMCSC method uses road geometry similarity and vehicle motion consistency to refine cross-domain spatial mapping, and the CATS-ICI strategy combines clue-aware trajectory matching with recursive state fusion. This design improves cross-domain association stability and reduces trajectory jitter during sensor handover.

(3) Experiments on a real highway section show that the proposed framework achieves a successful trajectory matching rate of 98.4% and a trajectory MAE of 0.23 m under the 452 vehicles/10 min test condition. The added ablation experiments further verify the contributions of radar–camera fusion, CIoU, OTTC, GMCSC, CATS, and ICI, demonstrating the effectiveness and originality of the proposed full-domain tracking framework.

Nevertheless, because OTTC mainly relies on motion-state prediction, future work will consider integrating occlusion-aware visual feature recovery to improve re-identification robustness under severe or long-duration occlusion [23].

## Figures and Tables

**Table 1:** Accuracy analysis of different detection methods.

Data Source	Field of View	$E_{radar}$ (%)	$E_{camera}$ (%)	$E_{fused}$ (%)
Low Traffic Volume	Near field	92.5	88.5	97.8
	Far field	88.6	81.5	95.4
Middle Traffic Volume	Near field	83.5	83.4	93.7
	Far field	85.2	80.2	92.8
High Traffic Volume	Near field	76.6	82.6	91.6
	Far field	80.3	78.8	89.2

**Table 2:** Ablation results of local tracking modules.

Method	Fusion	CIoU	OTTC	$E_{FP}$ (%)	$E_{IDSW}$ (%)	$E_{MOTA}$ (%)	$E_{MOTP}$ (%)
Radar-only + Deep-SORT	-	-	-	0.74	2.83	86.78	2.44
Camera-only + Deep-SORT	-	-	-	1.43	3.88	87.62	2.57
Fusion + Deep-SORT	+	-	-	0.43	1.36	90.74	0.71
Fusion + CIoU	+	+	-	0.35	0.82	91.63	0.56
Our Method	+	+	+	0.3	0.29	92.35	0.48

**Table 3:** Vehicle trajectory tracking results under different test conditions.

Test Set Size	452 Vehicle/10 min		745 Vehicle/10 min		956 Vehicle/10 min	
Proportion of trucks	15%	30%	15%	30%	15%	30%
Match (%)	98.4	96.5	95.3	92.4	93.1	90.9
MAE (m)	0.23	0.41	0.35	0.56	0.47	0.98

**Table 4:** Ablation results of cross-domain trajectory association modules.

Method	GMCSC	CATS	ICI	Match (%)	MAE (%)
Baseline stitching	-	-	-	84.7	1.42
*w*/*o* GMCSC	-	+	+	88.6	1.18
*w*/*o* CATS	+	-	+	90.3	0.92
*w*/*o* ICI	+	+	-	95.1	0.61
Our method	+	+	+	98.4	0.23

## Data Availability

The original contributions presented in this study are included in the article. Further inquiries can be directed to the corresponding author.
